# Recognizing Induced Emotions of Happiness and Sadness from Dance Movement

**DOI:** 10.1371/journal.pone.0089773

**Published:** 2014-02-24

**Authors:** Edith Van Dyck, Pieter Vansteenkiste, Matthieu Lenoir, Micheline Lesaffre, Marc Leman

**Affiliations:** 1 Faculty of Arts and Philosophy, Department of Arts, Music and Theater Sciences, Institute for Psychoacoustics and Electronic Music (IPEM), Ghent University, Ghent, Belgium; 2 Faculty of Medicine and Health Sciences, Department of Movement and Sports Sciences, Motor Control and Learning, Ghent University, Ghent, Belgium; VU University Amsterdam, Netherlands

## Abstract

Recent research revealed that emotional content can be successfully decoded from human dance movement. Most previous studies made use of videos of actors or dancers portraying emotions through choreography. The current study applies emotion induction techniques and free movement in order to examine the recognition of emotional content from dance. Observers (*N* = 30) watched a set of silent videos showing depersonalized avatars of dancers moving to an emotionally neutral musical stimulus after emotions of either sadness or happiness had been induced. Each of the video clips consisted of two dance performances which were presented side-by-side and were played simultaneously; one of a dancer in the happy condition and one of the same individual in the sad condition. After every film clip, the observers were asked to make forced-choices concerning the emotional state of the dancer. Results revealed that observers were able to identify the emotional state of the dancers with a high degree of accuracy. Moreover, emotions were more often recognized for female dancers than for their male counterparts. In addition, the results of eye tracking measurements unveiled that observers primarily focus on movements of the chest when decoding emotional information from dance movement. The findings of our study show that not merely portrayed emotions, but also induced emotions can be successfully recognized from free dance movement.

## Introduction

Emotions color all aspects of our daily life. They are essential to our social relationships, psychological well-being, cognitive functioning, moral sensitivity, and other important developmental processes [1]. Moreover, as they serve as signals that convey information about the friendliness or dangerousness of our environment, the communication and recognition of emotions has proved to be crucial to our survival [2]. In daily life, emotions can be communicated through speech prosody and voice quality [3]–[5]. However, they can also be transmitted through nonverbal and nonvocal communication channels. Theories of embodied cognition suggest that perceiving, experiencing, and thinking about emotions involves embodiment, or interaction between the emotional state of a person and his or her body [6]. Charles Darwin [7] himself introduced the idea that both humans and animals are capable of displaying emotions through motor behavior (especially posture). More recent research confirmed that emotions can for instance be recognized from facial expressions, which have distinct and universal expressive characteristics, signaling positive and negative feelings, attitudes and intentions [8]–[11]. Other studies have shown how emotions may successfully be recognized from bodily expression, especially from particular parts of the body such as the trunk [12], the arms [12], [13], and the hands [14]. In addition, both children and adults have been proved to master the ability to decode emotions from full body movements [14]–[20].

An interesting type of full body movement, in particular with regard to emotion research, is dance, which is one of the oldest forms of cultural expressions [21]. Moreover, dance is believed to facilitate the expression of several different emotions in a non-verbal way [22]. The ability to successfully decode emotions from dance movement is already present from early childhood onwards, as children as young as about five years old have proved to be able to recognize emotional meaning from dance. In addition, they have demonstrated to be capable of decoding the intensity of the emotions expressed by dancers when observing videos of adults moving expressively to music [15]. Some previous studies have used point-light displays, in which the body is represented by a small number of illuminated dots, positioned in such a way as to highlight the motion of the main body parts [13], [16], [18], [23], [24], while others addressed the issue of emotion recognition using full-light displays [15], [17]–[19], [23]. Emotion recognition by adults has proven to be more accurate from full-light than from point-light displays [18], which suggests a benefit from having the full complexity of the body shape available. Therefore, in the current study, videos of full-body movements of dancers are used instead of point-light displays.

Most previous research concerning emotion recognition from dance movement has used videos of actors or dancers portraying emotions such as happiness, sadness, fear, disgust, and anger [12], [13], [15], [17], [25], [26]. The assumption underlying the selection of emotions presented through acting in previous studies is that actors are typically believed to be experts in displaying emotional information corporeally and that acted emotions parallel emotions experienced in real life [14]. However, one might argue that these acted actions (either performed by professional or non-professional actors) are exaggerated and should rather be regarded as symbolic portrayals of the emotions at issue [18]. Moreover, a number of studies have revealed that not all actors generate equally identifiable, emotionally expressive dance movements [14], [26], [27]. Therefore, instead of considering emotions portrayed through acting, the current study applies emotion induction techniques in order to examine the recognition of emotions from dance movement by observers. In addition, contrary to previous studies, which have made use of choreography to study expressive dance movements [24]–[27], the dancers in this study were able to move freely as we believe that this facilitates more accurate expression of emotion. Moreover, this method could prove to be a more ecologically valid approach for studying emotion recognition from dance movement.

In a previous study by Van Dyck et al. [28], which examined the impact of induced emotions (happiness and sadness) on dance movements of participants dancing freely to emotionally neutral music by measuring and analyzing the kinematics of the movements, evidence of the effect of emotion induction on dance movement was indeed provided. In the current study, we examine whether observers are also able to decode induced emotions from free dance movement. We expect that observers are capable of distinguishing between happy and sad dances. We also presume that female observers perform better in the recognition task than their male counterparts, as women are believed to be superior in understanding others' emotions [29]–[31]. In addition, as women generally experience and express emotions more intensely than do men [32], we expect the observers to have a higher success rate when judging the emotional state of female than of male dancers. Van Dyck, et al. [28] showed that movements of participants who danced after being induced to feel emotional states of happiness proved to be faster, more accelerated, more expanded and more impulsive than after sad emotion induction. Thus, we also presume that observers will label the kinematics of the dances in a similar way. While the recognition of emotions from dancers' movements has received considerable attention, little is known about visual search patterns people use to gather information on which this recognition is built. Therefore, in this study, we use the behavioral method of eye tracking to examine where observers direct their focus. As Van Dyck et al. [28] revealed that the differences between the emotion conditions were primarily detectable from hand movement, we expect observers to mainly focus on the gestures of the dancers' hands.

## Methods

### Ethics Statement

The study was approved by the Ethics Committee of the Faculty of Arts and Philosophy of Ghent University. In addition, all participants signed a form to declare that they participated voluntarily, that they had received sufficient information concerning the tasks, the procedures, and the technologies used, that they had the opportunity to ask questions and that they were aware of the fact that recordings of eye movements were made, for scientific and educational purposes only.

### Observers

The sample of observers concerned a non-random selection. The selection criterion was based on the condition of being aged between 20 and 35 years of age, as it is known that emotion recognition is not fully matured until early teenage years and it is believed that the duration of negative emotional states decreases with age [33]. In addition, observers were selected based on their gender and their record of music and dance training (both males/females; with/without music training; with/without dance training). Thirty adult observers (15 females, 15 males) took part in the study. The average age of the observers was 27.23 years (*SD*  = 3.43). 60% had received musical training, and of those, the average time spent in musical training was 6.23 years (*SD*  = 6.70). About half of the observers (56.70%) had also trained in dance and spent, on average, 2.67 years (*SD*  = 4.02) in dance training. In addition, 90% of the observers reported that dancing is an activity that forms a part of their lives, with varying degrees of frequency (13.30% danced about once a year; 63.30% danced about once a month; 10% danced about once a week; 3.30% danced more than once a week). As participants were both recruited by the Department of Arts, Music, and Theater Sciences and the Department of Movement and Sports Sciences of Ghent University, Belgium, a heterogeneous mix of people experienced in judging expression related to their training was obtained; e.g., physical activity and action observation (from sports/dance training) and a specific talent in judging dance movement (from music/dance training). The observers received no compensation for participating in the study.

### Stimuli

**Figure 1:**
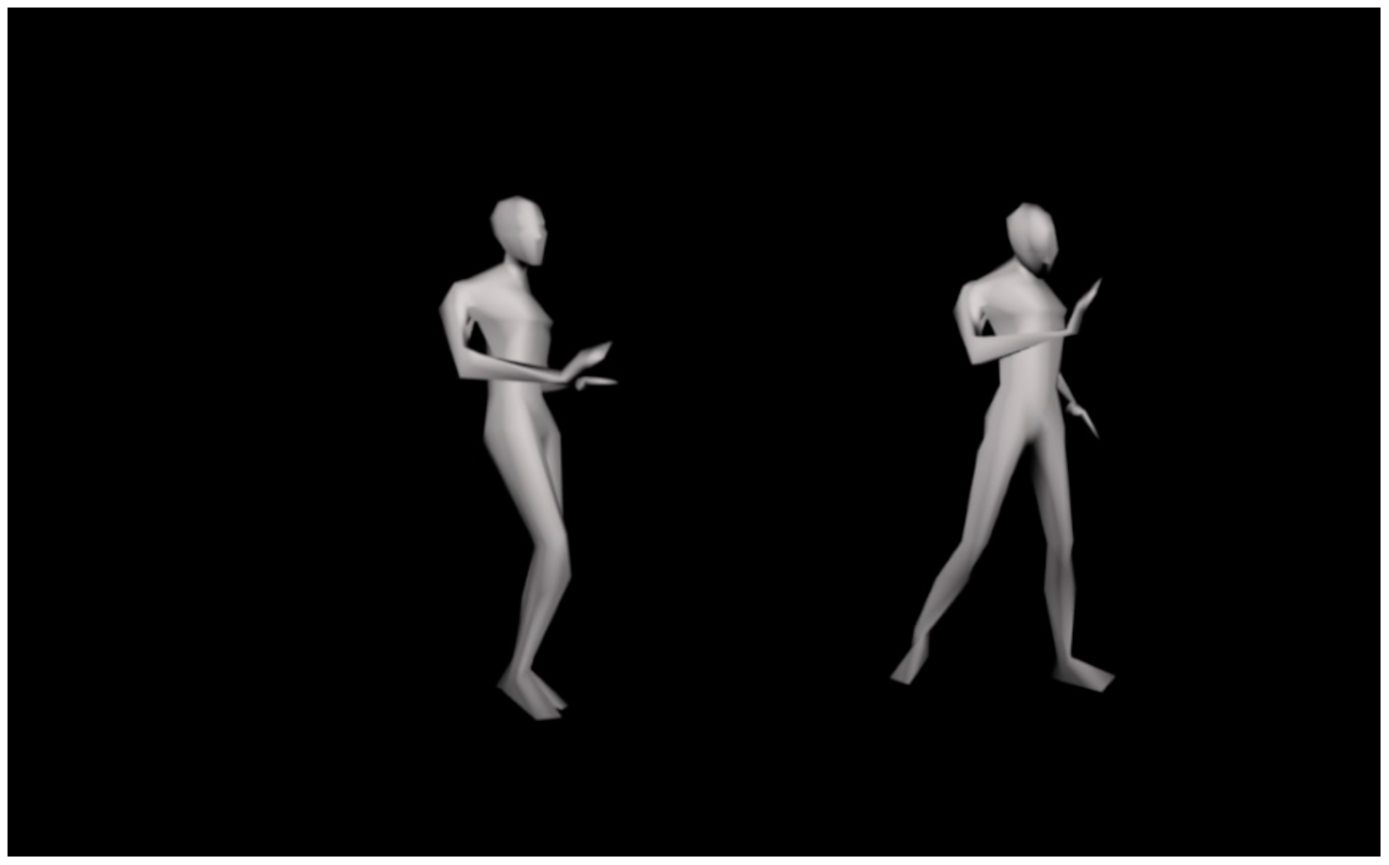
Example of a video clip with a pair of dance performances of the same dancers; one in the happy condition and one in the sad condition.

Sixteen video clips were used in this study. The video clips were recorded with motion capture cameras in a previous study [28], in which the effect of induced emotions on the kinematics of dance movements was studied. In that particular study, participants were induced to feel emotional states of either happiness or sadness and then danced intuitively to an emotionally neutral piece of music. Each participant performed the dance both in the happy and sad condition. For the current study, silent video clips showing depersonalized, androgynous avatars were created from the movement data of the non-expert dancers. Each of the 16 video clips consisted of a pair of dance performances that were presented side-by-side and were played simultaneously; one of a dancer in the happy condition and one of the same dancer in the sad condition (see Fig. 1). Three series of video clips were prepared, each time with randomized order of the clips. Moreover, the order of the emotion conditions (whether happy and sad dances were presented on the left or right side in the video clips) was randomized. Half of the dancers were female and all emotional states were presented an equal number of times on the left and on the right side of the screen. All video clips were edited to exactly 10 seconds in length. Three practice clips preceded the 16 clips to be rated. The practice clips consisted of video clips that were not presented in the actual series of clips (e.g., with a different order of the emotion conditions). The video clips were presented on a 22-inch computer monitor. The distance between the monitor and the participants was about 27.60 inch and the stimuli occupied approximately 37.8° of the field of view.

### Eye Tracking

The behavioral method of eye tracking was chosen as it concerns an unobtrusive strategy to gather information with regard to the specific focus of the observers. It consists of a more implicit measure of what is guiding the gazer's motivation than for instance self-reports, and offers quantitative evidence of the observer's visual and attention processes. In addition, eye movements are the result of the interaction between cognitive and perceptual processes. Therefore, we believe that eye tracking can serve as an adequate tool to investigate the process of emotion recognition.

Eye movements were recorded using a Remote Eye tracking Device (RED) by SensoMotoric Instruments (SMI), which operated at 120 Hz. A five-point calibration was used and two validations were performed during the experiment. To calculate the time participants watched the different regions of the body, dynamic Areas Of Interest (AOIs) were coded on the video clips using BeGaze 3.2 (SMI). Once the AOIs were coded, the dwell-time percentages (percentage of time the eyes were directed towards the AOI) were retrieved. In order to control for AOI size, dwell-time percentages were normalized for the size of the specific body area. All clips were coded with following AOIs: head, chest, arms, hands, hips, legs, and feet.

### Procedure

Observers were asked to watch the three practice clips and 16 dance videos and to fill out a short questionnaire after each video. In the questionnaire, they were asked to identify which of the two dance performances was happy and which was sad. The observers were aware of the fact that the same dancer, in one excerpt feeling happy, in the other feeling sad, executed each of the two simultaneously presented dance performances in a trial. Moreover, the observers were asked to rate each dance performance concerning its kinematic properties. They rated their impressions with regard to the features velocity, acceleration, impulsiveness, and expansion on a five-point Likert scale (‘1 =  Not at all’, ‘2 =  Slightly’, ‘3 =  Moderately’, ‘4 =  Very’, ‘5 =  Extremely’). At the end of the experiment, the observers were asked to fill out a second type of questionnaire, which contained questions concerning their music and dance background.

### Data availability

Data will be made freely available upon request.

## Results

### Emotion recognition

A one-sample chi-square test revealed that, overall; observers were able to recognize the correct emotion from the dance movements, *χ* ^2^ (1)  = 255.21, *p*<.001. This represents the fact that, based on the odds ratio, the odds of observers recognizing the correct emotion were 6.38 times higher than making an incorrect decision concerning the emotional state of the dancers. In addition, the effect of the order of the videos and the order of the emotions (either left or right), but also of the sex of the dancers and the observers was examined. Pearson's chi-square tests (using Yates' continuity correction for 2×2 contingency tables) revealed no significant associations between the order of the videos, *χ* ^2^ (2)  = .64, *p* = .89, the order of the emotions, *χ* ^2^ (1)  = 1.78, *p* = .18, or the sex of the observers, χ^2^ (1)  = 1.14, *p* = .29. However, there was a significant association between the sex of the dancers and the results of the emotion recognition task, *χ* ^2^ (1)  = 28.47, *p*<.001. Based on the odds ratio, the odds of recognizing the correct emotional state were 5.38 times higher when observers were watching female dancers than when they were observing male dancers. An overview of the results of the emotion recognition task (presented in percentages) is shown in Table 1.

**Table 1:** Observers' responses to the emotion recognition task.

	Correct answers on the emotion recognition task	Incorrect answers on the emotion recognition task
**Overall emotion recognition**	86.46%	13.54%
**Effect order videos**		
Order 1	28.13%	5.21%
Order 2	29.17%	4.16%
Order 3	29.17%	4.16%
**Effect order emotions**		
Happy – Sad	42.08%	7.92%
Sad – Happy	44.38%	5.62%
**Effect sex observers**		
Male	44.17%	5.83%
Female	42.29%	7.71%
**Effect sex dancers**		
Male	38.96%	11.04%
Female	47.50%	2.50%

### Ratings of kinematic features

In order to check whether observers associated particular kinematic features with specific emotional states, rating data for the different movement features were compared between the two emotion conditions by means of Wilcoxon signed-rank tests. However, no significant differences were found between the happy and sad performances for either velocity, *Z*(479)  = −0.39, *p* = .70, acceleration, *Z*(479)  = −0.39, *p* = .70, impulsiveness, *Z*(479)  = −0.94, *p* = .35, or expansion, *Z*(479)  = −0.88, *p* = .38.

### Eye tracking

Validation tests showed an average accuracy (i.e., the angular average distance from the actual gaze point to the one measured by the eye tracker) of 0.37° (*SD*  = 0.12) and the average tracking ratio (% of time eye movement was actually measured) was 89.90% (*SD*  = 3.70). Eye tracking data of all participants proved to be accurate enough in order to be analyzed statistically.

**Figure 2:**
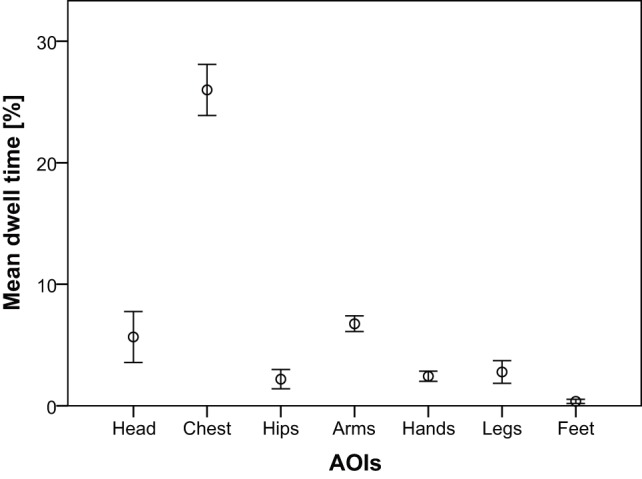
Mean dwell time for the AOIs. Data presented are mean ± *SE*.

AOIs were analyzed in order to check the focus of the observers' gaze. A Kolmogorov-Smirnov test (KS-test) showed that the assumption of normality could not be accepted. A Friedman's ANOVA with the AOIs as test variables showed a significant difference in mean dwell time between the different AOIs, *χ* ^2^(6)  = 128.69, *p*<.01. Wilcoxon tests were used to follow up this finding. A Bonferroni correction was applied and so all effects are reported at a.00048 level of significance. It appeared that the mean dwell time was significantly higher for the chest than for the head, *Z*(29)  = −4.76, hips, *Z*(29)  = −4.78, arms, *Z*(29)  = −4.78, hands, *Z*(29)  = −4.78, legs, *Z*(29)  = −4.78, and feet, *Z*(29)  = −4.78. In addition, mean dwell time was significantly higher for the arms than for the hips, *Z*(29)  = −4.68, hands, *Z*(29)  = −4.78, legs, *Z*(29)  = −4.39, and feet, *Z*(29)  = −4.78. Finally, mean dwell time was significantly lower for the feet than for the head, *Z*(29)  = −4.33, chest, *Z*(29)  = −4.78, hips, *Z*(29)  = −4.61, arms, *Z*(29)  = −4.78, hands, *Z*(29)  = −4.70, and legs, *Z*(29)  = −4.78. An overview of the mean dwell time for the different AOIs is presented in Figure 2.

**Figure 3:**
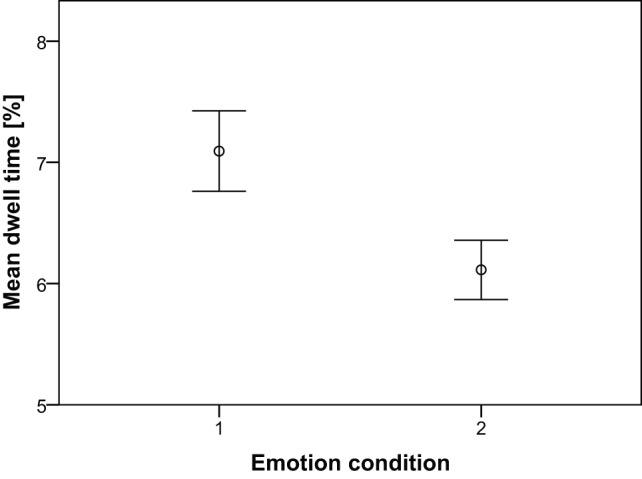
Mean dwell time for the emotion conditions. Data presented are mean ± *SE*.

In addition, the effect of the emotion condition on the focus of the observers' gaze was investigated. A KS-test showed that the mean dwell time was significantly normal, *D*(30)  = 0.11, *p*>.05, and a Levene's test revealed that the variances were equal for the happy and sad condition, *F*(1, 58)  = 2.58, *p*>.05. A dependent *t*-test with the mean dwell times for each condition as test variables showed that, on average, dwell times were significantly higher in the happy condition (*M* = 7.09, *SE*  = .89) than in the sad condition (*M* = 6.11, *SE*  = .66), *t*(29)  = 5.29, *p*<.001. An overview of the mean dwell time for the different emotion conditions is presented in Figure 3.

## Discussion

In this experiment, it was investigated whether observers are able to decode emotional content from corporeal articulations of dancers moving to an emotionally neutral piece of music after emotions of either happiness or sadness had been induced. The results revealed that observers were indeed capable of successfully recognizing the intended emotion from the dance movements. This resonates with findings of previous research on emotion recognition from dance movement using portrayed emotions. Boone and Cunningham [15], Shikanai et al. [19], and Montepare et al. [26] for example, showed that emotions such as happiness, sadness and anger expressed in dance could be accurately identified by observers. Therefore, our results are in accordance with findings of previous studies, but also extend them by showing that, in addition to portrayed, or ‘acted’, emotions, induced emotions of happiness and sadness can also be decoded from unchoreographed dance movements by observers.

As it is well documented that, generally, women are better at understanding and considering the feelings and needs of others compared to men [29]–[31], we presumed female observers to recognize the induced emotions more often than their male counterparts. However, our results did not support this premise and apparently this finding is not all that unique. A similar study by Ross et al. [18] regarding emotion recognition from body movements of actors portraying emotions of happiness, sadness, fear, and anger, for instance, did not reveal any sex effects with regard to the observers either.

On the other hand, in the current study, a significant association with recognition accuracy was unveiled regarding the sex of the dancers, as the emotional state was more often recognized for female dancers than when male dancers were being observed. This suggests that women are more proficient in expressing their personal feelings in a corporeal manner compared to men. This is in accordance with a fairly substantial body of research, which has demonstrated that women are more emotional, and both experience and express emotions more intensely than men [32], [34].

By capturing and analyzing the kinematics of the movements of participants dancing to an emotionally neutral stimulus, Van Dyck et al. [28] unveiled that, after happy emotion induction, movements proved to be faster, more accelerated, more expanded (calculated as the distance between joints of the upper limbs and the body center), and more impulsive (calculated as the amplitude range of the velocity curve divided by the duration of a motion segment) than after sad emotion induction. Likewise, similar findings were reported in previous studies regarding the effect of portrayed emotions of joy, grief, anger, and fear on dance movement. Both Lagerlöf and Djerf [17] and Camurri et al. [25] showed that the duration of grief performances was remarkably longer and consisted of a smaller amount of tempo changes compared to performances in other emotion conditions. In addition, movements were more contracted in the grief and fear conditions than in the joy condition. Finally, Camurri et al. [25] revealed a main effect for Quantity of Motion (a measure of the total amount of detected motion, involving velocity and force), as performances in the joy condition received higher mean scores than those in the grief condition. However, in the current study, which relied on video data of the study of Van Dyck et al. [28], no significant differences between the emotional states were unveiled with regard to observers' designation of kinematic properties. This could be due to the choice of the specific kinematic labels (e.g., acceleration, expansion, etc.), which might have proved to be too abstract for the observers to fully comprehend. Another explanation could be found in the short duration (10 seconds) of the film clips, which might not have enabled thorough examination of the kinematic characteristics of the dance movements. However, a number of previous studies regarding emotions expressed through body movement have demonstrated that observers require only a limited time to analyze kinematic characteristics rigorously [18], [23], [26], [35], [36]. A novel study with a new set of kinematic properties comparing dance clips of different lengths might investigate the significance of the specific labels and the duration of dance stimuli more thoroughly.

As the hands are generally believed to have a privileged role in music-related gestures [37], and as Van Dyck et al. [28] obtained more significant differences for the hands than for any other body part, we expected that observers would mainly focus on the gestures of the dancers' hands. However, eye tracking data from the current study unveiled a specific focus of the observers on the chest area. Although this finding did not fit our expectations, several explanations arise from the data. First, most of our body movements tend to start from the more proximal segments and develop towards the more distal limbs [38]. This implies that information on changes in movement direction or acceleration might be readily seen in the trunk/shoulder area first. Second, even though the observers' general focus is on the chest area, gestural information concerning other body parts is not necessarily disregarded as the participants are still capable of perceiving movements of other parts of the body, in relation to the chest. In this specific experimental set-up, most of the movements of the arms and legs were still within the useful field of view when focusing on the chest. The reported gaze behavior therefore suggests that dancers' emotions were analyzed using an extended visual span and parafoveal processing. This visual strategy has been described in fields such as sports [39], [40] and radiology [41] and has been labeled as ‘the holistic model of image perception’. This visual strategy suggests that observers do not pay attention to the head, the hands, the hips, etc. as separate parts of the body, but rather see the human body as one entity. Our finding with regard to the focus on the chest accords with previous research on emotion and body posture as several studies have emphasized the importance of the posture and position of the torso as indicators for emotional content. Schouwstra and Hoogstraten [42], for instance, used stick drawings of armless figures and varied the positions of the spine (and head). Their study revealed that upright postures were judged more positively, and forward leaning postures more negatively. In addition, an unpublished study by Inouye [43], which required participants to pose a wooden artist's doll, suggested that basic emotions could be represented in terms of sagittal movement, spinal flexion, open/closed and forwards/backwards reaching, and facial orientation towards or away from the eliciting stimulus.

An additional finding with regard to the eye tracking data is that observers focused more on the dancers in the happy condition than on those in the sad one. This result could be explained by the idea of ‘salience’. Gazing allocation research has shown that observers' attention is generally allocated to the most salient locations in a scene, or the locations that stand out relative to their neighbors [44]. As Van Dyck et al. [28] unveiled that, after happy emotion induction, movements proved to be faster, more accelerated, more expanded, and more impulsive than after sad emotion induction, the bodies of dancers in the happy condition were in all probability more salient than those of dancers in the sad one, and therefore, attracted more attention.

Previous research revealed that, even though emotion recognition is not fully matured until early teenage years [45], [46] children as young as about five years old perform above chance in successfully perceiving emotional information from body language [15], [18]. Moreover, they are capable of decoding the intensity of the emotions expressed by dancers when observing videos of dancers moving to music [15]. As it is believed that the duration of negative emotional states decreases with age [33], the current study only considered a specific age group (all participants were between 24 and 34 years of age). However, a future study could investigate whether also children or observers in other age groups are capable of successfully recognizing induced emotions from free dance movements.

One could argue that movement ratings and emotion classifications might have influenced each other. Possibly, observers distinguished dance movements on the basis of kinematic characteristics rather than emotional content. On the other hand, since observers first judged the emotional quality of the dance performances and only rated the kinematics later, the emotion classification could have affected kinematic appraisal. However, in this study, no differences were found between the happy and sad performances for any of the kinematic features. Notwithstanding, future research could explore the link between kinematics and emotion classification more closely. Moreover, if a link could be found, it could be explored whether it concerns a causal one.

With regard to the stimuli used in this study, each film clip consisted of two dance performances executed by the same individual. Pairs of performances of one and the same dancer were employed in order to ensure optimal control over possible confounding effects caused by characteristics of the dancers, such as the their personality [47], gender [48], [49], and proclivity to dance [50], since these features have been proved to influence human dance movement significantly. However, the skill to discriminate between happy and sad performances of the same dancer might be different from the ability to differentiate between happy and sad dances across different performers. Yet, this is a matter of some speculation and would benefit from further study.

Another deliberate choice in our experimental setup was the employment of free dance movement. This type of dance movement was selected because of its ecological validity and our results suggest that it can indeed serve as a suitable alternative to choreography in research on emotion recognition. Participants were allowed to move intuitively, so they might have felt less inhibited and as a result moved in a less restricted manner. Additionally, the removal of the limitations of a prescribed dance routine has the advantage of opening participation in the study to people without a professional background in dance, thus enabling researchers to address a far broader group of potential ‘dancers’ in future studies regarding emotion recognition.

### Conclusion

In summary, this experimental study examined whether observers are able to recognize induced emotions from free dance movement. Our results are in tune with results of similar studies, but they also extend previous research, showing that, in addition to portrayed emotions, also induced emotions can be perceived from unchoreographed dance movement by adult observers. Moreover, this study shows that female dancers are better at communicating emotional meaning corporeally than their male counterparts. Finally, the results of this study unveiled that observers generally focus on movements of the chest when decoding emotional information from dance movement.
